# Detection of *Fusarium solani* using cutinase antibody and its application in diagnosing fungal keratitis in an animal model

**DOI:** 10.1371/journal.pone.0330455

**Published:** 2025-08-20

**Authors:** Hye-Jeong Jo, Min-Jeong Kim, Hae-Ahm Lee, Fu-Shi Quan, Hyun-Hee Kong, Eun-Kyung Moon

**Affiliations:** 1 Department of Biomedical Science, Graduate School, Kyung Hee University, Seoul, Republic of Korea; 2 Department of Parasitology, School of Medicine, Chungbuk National University, Cheongju, Republic of Korea; 3 Department of Medical Zoology, School of Medicine, Kyung Hee University, Seoul, Republic of Korea; 4 Medical Research Center for Bioreaction to Reactive Oxygen Species and Biomedical Science Institute School of Medicine, Graduate School, Kyung Hee University, Seoul, Republic of Korea; 5 Department of Parasitology, College of Medicine, Dong-A University, Busan, Republic of Korea; Leibniz-Institut fur Naturstoff-Forschung und Infektionsbiologie eV Hans-Knoll-Institut, GERMANY

## Abstract

Fungal keratitis (FK) is a sight-threatening infectious disease that can result in blindness if not appropriately treated. Although keratitis associated with filamentous fungi was rarely reported in the past, the incidence of fungal keratitis due to contact lens usage has increased. The clinical manifestations of fungal keratitis often resemble those of other corneal infections, potentially delaying accurate diagnosis and treatment. In this study, we developed a *Fusarium*-specific polyclonal peptide antibody targeting the cutinase of *F. solani* and evaluated its diagnostic potential for FK in an animal model. To assess the specificity of the cutinase antibody, we employed enzyme-linked immunosorbent assay (ELISA). The ELISA results demonstrated that the cutinase antibody specifically interacted with the cell lysates and conditioned media of *F. solani*. Additionally, an immunocytochemistry assay confirmed the specificity of the cutinase antibody for *F. solani* in samples co-cultured with human corneal epithelial (HCE) cells and other keratitis-causing agents. To validate our in vitro findings, FK animal models were established by infecting the corneas of BALB/c mice with *F. solani*. The cutinase antibody specifically detected *Fusarium* antigens in the tear-wash samples and eyeball lysates of FK mice. These results demonstrate that the cutinase antibody is highly specific to *F. solani* antigens, indicating its potential utility in developing an antibody-based diagnostic method for FK.

## Introduction

*Fusarium solani* is ubiquitously distributed in the environment and is frequently identified, particularly in soil samples [1–3]. It functions primarily as a plant pathogen, inflicting significant agricultural damage, yet it is also capable of infecting humans, where it is a major etiological agent of keratitis, dermatitis, and other conditions [3–5]. Notably, *F. solani* is the predominant cause of fungal keratitis (FK), a rare yet increasingly prevalent ocular disease globally [6]. FK arises when the fungus infects a compromised cornea. Traditionally, FK has been prevalent among agricultural workers due to corneal injuries from crops, however, the advent and widespread use of contact lenses have contributed to an increased incidence within the broader population [1]. Without treatment, the fungal infection may infiltrate deeper ocular layers, potentially resulting in endophthalmitis and, in severe instances, blindness or enucleation [7]. However, early diagnosis of fungal keratitis presents a significant challenge due to its initial symptoms such as eye pain (ophthalmalgia), blurred vision, and excessive tearing (epiphora) which are similar to those of other forms of keratitis, including *Acanthamoeba* keratitis (AK) [8]. This similarity can make it difficult to accurately and promptly diagnose the specific type of keratitis, underscoring the need for specific and sensitive diagnostic tools or methods.

Current diagnostic methods, such as direct microscopic examination, polymerase chain reaction (PCR), in vivo confocal microscopy (IVCM), and culture, each present specific limitations [9]. These limitations include the discomfort associated with obtaining samples through corneal scraping, the potential for false-positive results, and the requirement for expert interpretation [5,9–11]. Although eye tissue culture is widely regarded as the gold standard, it is time-consuming and may result in diagnostic delays [7].

In our previous study, we developed antibodies targeting proteins secreted by *A. castellanii*, such as ACAP, PBP, CE, and CM, to facilitate the diagnosis of AK [12–15]. These antibodies successfully detected *A. castellanii* in a mouse model of AK, highlighting the potential for a rapid and non-invasive diagnostic approach for infectious keratitis. Concurrently, murine monoclonal antibodies and polyclonal antibodies were generated against purified cutinase from the culture fluids of *F. solani* [16]. All these antibodies recognized cutinase from plant pathogens including *F. solani*, *Botrytis cinerea*, *Blumeria graminis*, and *Puccinia* species. This demonstrates the potential for developing cutinase antibodies to be used in diagnosing FK.

Cutinase is an enzyme responsible for hydrolyzing ester bonds in cutin, a major constituent of plant cell walls, and is predominantly secreted by fungi and bacteria. Although bacterial and fungal cutinases exhibit similar substrate specificity and catalytic properties, they differ in their amino acid composition [17]. Based on these findings, we hypothesized that antibodies against cutinase, a protein secreted by *F. solani*, could also detect *F. solani* antigens in FK samples. By creating antibodies specific to cutinase, it may be possible to accurately detect fungal infections, distinguishing them from other types of keratitis with similar symptoms. This approach could lead to more precise and timely treatment options for patients.

This study explores the utilization of a cutinase antibody for the detection of *Fusarium* spp., with a focus on its application in diagnosing fungal keratitis within an animal model. The specificity and sensitivity of the cutinase antibody highlight its potential as a diagnostic tool, offering a promising approach for the early and accurate detection of fungal keratitis caused by *Fusarium* spp.

## Materials and methods

### Cell cultures

*Fusarium solani* (NCCP 32678), *Pseudomonas aeruginosa* (NCCP 16091), and *Staphylococcus aureus* (NCCP 15920) were procured from the Korea Centers for Disease Control and Prevention (Osong, Republic of Korea). *Acanthamoeba castellanii* (ATCC 30868) and human corneal epithelial (HCE) cells (ATCC PCS-700–010) were acquired from the American Type Culture Collection (Manassas, VA, USA). *F. solani* was cultivated in Sabouraud Dextrose (SD) medium at 37°C, while *A. castellanii* was maintained in peptone-yeast-glucose (PYG) medium at 25°C. HCE cells were aseptically cultured in keratinocyte growth medium (KGM BulletKit™; Lonza, Portsmouth, NH, USA) under conditions of 37°C with 5% CO_2_. Both *P. aeruginosa* and *S. aureus* were grown in Brain Heart Infusion (BHI) medium at 37°C. The cell lysates were prepared by suspending collected cells in PRO-PREP™ Protein Extraction Solution (iNtRON Biotechnology, Seongnam, Republic of Korea) followed by sonication. The conditioned media were prepared by culturing cells for 5 days, followed by centrifugation to collect the supernatant.

### Polyclonal peptide antibody production of cutinase protein

The peptide sequence of the cutinase protein utilized as the immunogen was NH2-SIEDLDSAIRDKIAG-COOH. Two New Zealand White rabbits were intradermally immunized with 1.0 mg of peptide-keyhole limpet hemocyanin (KLH) conjugates in complete Freund’s adjuvant on day 0, followed by 0.5 mg of the peptide in incomplete Freund’s adjuvant on days 21, and 35. Following the second immunization, the antisera titer was evaluated using an indirect ELISA, employing peptide-bovine serum albumin (BSA) conjugates as coating antigens, until the titer reached a plateau. After the final immunization, blood samples were collected via cardiac puncture from each rabbit. All experimental procedures were conducted by AbFRONTIER (Seoul, Republic of Korea).

### Enzyme-linked immunosorbent assay (ELISA)

*Fusarium-*specific cutinase antibody responses were quantified using an enzyme-linked immunosorbent assay (ELISA). The serum was diluted from 1:5–1:5,000, and antigen was serially diluted from 100 μg/ml to 0.001 μg/ml, each in 10-fold steps. Cell lysates and conditioned media from *F. solani*, *A. castellanii*, HCE cells, *P. aeruginosa*, and *S. aureus* were utilized as coating antigens. Ninety-six-well plates were coated with the antigen solution in a carbonate buffer (0.1 M sodium carbonate, pH 9.5) and incubated overnight at 4°C. After each incubation step, the plates were washed three times with PBST. Blocking was performed with 0.2% gelatin at 37°C for 2 h. After blocking, the serum was added and incubated at 37°C for 1 h. Subsequently, horseradish peroxidase (HRP)-conjugated goat anti-rabbit IgG (Cusabio Co. Ltd, Wuhan, China) at a dilution of 1:2,000 was added and incubated at 37°C for 1 h. All antibodies were prepared in PBST. O-Phenylenediamine (OPD; Zymed, San Francisco, CA, USA) was dissolved in citrate-phosphate buffer (pH 5.0) containing 0.03% H_2_O_2_ and used as a substrate. Absorbance at 450 nm was measured using the EZ Read 400 microplate reader (Biochrom Ltd., Cambridge, UK). Sera from non-immunized rabbits served as the negative control.

### Immunocytochemistry (ICC)

HCE cells (3 × 10^5^ cells/well) were cultured on sterile cover glass in a 6-well plate and incubated at 37°C with 5% CO_2_ for 24 h. The following day, *A. castellanii* (5 × 10^5^ cells/well) was added and incubated for 5 h. *F. solani*, *P. aeruginosa*, and *S. aureus* were cultured in liquid broth to the early exponential phase (OD_600_ = 0.8) and subsequently co-cultured with the HCE cells and *A. castellanii* for 1 h. Following incubation, the culture media was discarded, and the cells were washed with phosphate-buffered saline (PBS) and fixed with ice-cold 100% methanol for 5 min at room temperature (RT). After repeated washing with PBS for 5 min, the cells were incubated in a blocking buffer (1% BSA and 22.5 mg/ml glycine in PBST) for 30 min at RT. The cells were then incubated overnight at 4°C with cutinase polyclonal antibodies in the blocking buffer (1:200) and subsequently probed with anti-rabbit IgG conjugated with fluorescein isothiocyanate (FITC) (1:400) for 2 h. Following three washes with PBS, the cells were stained with VECTASHIELD mounting medium (Abcam, Cambridge, UK) and observed under a fluorescence microscope (Leica DMi8, Wetzlar, Germany).

### Animals and ethics requirements

All procedures involving animals were conducted in adherence to the Animal Research: Reporting of *In Vivo* Experiments (ARRIVE) guidelines. The experimental protocols involving animals received approval from the Institutional Animal Care and Use Committee of Kyung Hee University Medical Center (KHSASP-24–375). Animals were accommodated in certified facilities, adhering to 12 h day and night cycle with unrestricted access to nourishment and hydration. Appropriate methods were employed to ensure ethical standards, and every effort was made to minimize both the number of animals used and their suffering. The mice used in the experiment were euthanized in a carbon dioxide chamber before tissue sampling.

### Production of *Fusarium* keratitis (FK) animal model

Ten 8-week-old BLB/c mice were purchased from NARA Biotech (Seoul, Republic of Korea). Five mice were used as the negative control group, and the other five were used as the FK animal model group. *F. solani* cultures were centrifuged at 3,000 rpm for 10 min to obtain a concentration of 1 × 10^6^ cells. A commercial soft contact lens (Proclear 1-Day Contact Lenses, CooperVision, NY, USA) was sectioned into 2 mm circles using a hole puncher. The cell pellet was suspended in 20 μl of sterile PBS and gently overlaid on the surface of the contact lens, followed by incubation at 25°C for 1 h. Mice were anesthetized via intraperitoneal injection of avertin (250 mg/kg) (Sigma-Aldrich, St. Louis, MO, USA), and multiple incisions were made on the corneal surface using a 25-gauge syringe needle. Following corneal abrasion, contact lenses containing *F. solani* were carefully positioned on the ocular surface, and the eyelids were sutured using 6–0 nylon sutures (Woorimedical, Namyangju, Republic of Korea). The mice’s eyes were monitored daily for 10 days to evaluate keratitis development. On the 10th day post-infection, the sutures were removed, and tear-wash samples as well as whole eyeballs were collected. Tear-wash samples were collected by washing each *F. solani*-inoculated eye with 10 μl of cold sterile PBS, resulting in a total collection volume of 100 μl, and the whole eyeball was homogenized in 500 μl of sterile PBS. Naïve mice were also sampled and served as negative controls. All samples were promptly frozen and stored at −80°C until further use.

### Detection of *F. solani* antigen in FK mouse model using cutinase antibody

The capability of cutinase antibodies to detect *F. solani* antigens in the FK mouse model was assessed using an ELISA assay. Tear-wash samples (50 μg/ml) and whole eyeball lysates (50 μg/ml) were utilized as antigens, coated on a 96-well plate using a carbonate coating buffer, and incubated overnight at 4°C. Subsequently, the plates were washed with PBST and blocked with 0.2% gelatin at 37°C for 2 h. Following additional washing with PBST, undiluted cutinase antibody was added to each well and incubated at 37°C for 1 h. Unimmunized rabbit serum served as the negative control group. Horseradish peroxidase (HRP)-conjugated goat anti-rabbit IgG (Cusabio Co. Ltd, Wuhan, China) was then added at a dilution of 1:2,000 and incubated at 37°C for 1 h. The OPD substrate was dissolved in citrate-phosphate buffer containing 0.03% H_2_O_2_. The optical density at 450 nm was measured using a microplate reader (Biochrom Ltd., Cambridge, UK).

### Statistical analysis

All experimental data were statistically analyzed using GraphPad Prism Software version 8.0 (San Diego, CA, USA), and group comparisons were conducted using Student’s *t-*test (two-tailed). Data are presented as mean ± standard deviation (SD). Statistical significance between groups is denoted by asterisks, with *P* values less than 0.05 considered statistically significant (*P < 0.05, **P < 0.01, ***P < 0.001, and ****P < 0.0001).

## Results

### Identification and sequence analysis of cutinase from *F. solani*

The cutinase protein of *Fusarium solani* consists of 693 base pairs, encoding 230 amino acids with a calculated molecular mass of 25.3 kDa (GenBank accession number AAA33334.1). To evaluate the potential of the cutinase antibody to differentiate between various causes of keratitis, a comparative analysis of amino acid sequences was conducted using BLASTP. The protein homology search revealed that the cutinase of *F. solani* shares 33.82%, 21.24%, and 20.75% similarity with the cutinase of *Aspergillus fumigatus* (GenBank accession number XP_755775.1), *Pseudomonas aeruginosa* (GenBank accession number AXL68988.1), and *Staphylococcus aureus* (GenBank accession number RZI07573.1), respectively (Table 1).

**Table 1:** Identification of cutinase amino acids as determined by BLASTP.

Name	Accession No.	Length (aa)	Identities (%)
F.s_cut^a^	AAA33334.1	230	100
A.f_cut^b^	XP_755775.1	375	33.82
P.a_cut^c^	AXL68988.1	427	21.24
S.a_cut^d^	AXL68988.1	160	20.75

^a^*F. solani*_cutinase (F.s_cut).

^b^*A. fumigatus*_cutinase (A.f_cut).

^c^*P. aeruginosa*_cutinase (P.a_cut).

^d^*S. aureus*_cutinase (S.a_cut).

**Fig 1:**
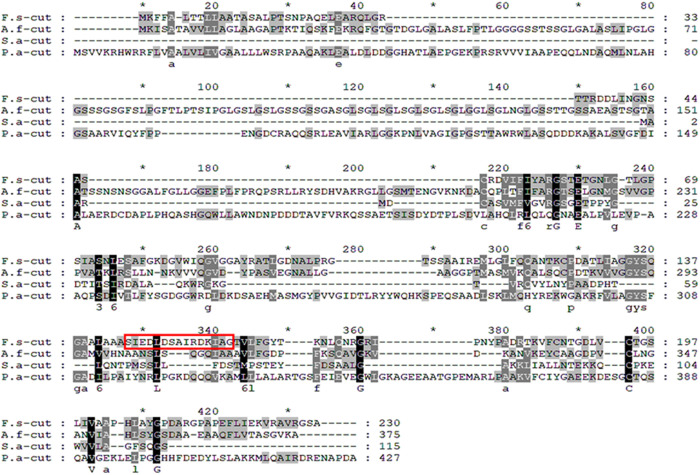
Amino acid sequence alignments of cutinases from various organisms. The aligned cutinase amino acid sequences of *Fusarium solani* (F.s_cut, AAA33334.1), *Aspergillus fumigatus* (A.f_cut, XP_755775.1), *Pseudomonas aeruginosa* (P.a_cut, AXL68988.1), and *Staphylococcus aureus* (S.a_cut, RZI07573.1) were compared. Multiple sequence alignment was performed using ClustalX software, with sequence conservation visualized through differential shading. A red boxed sequence from this alignment was employed in the production of the anti-cutinase polyclonal antibody.

Based on amino acid homology, secondary structure analysis, hydrophobicity, and antigenicity profiles, the region spanning amino acids 145–159 was identified as the optimal antigenic site for the production of the cutinase antibody (Fig 1, red boxed area).

### Antibody responses to cutinase using ELISA

**Fig 2:**
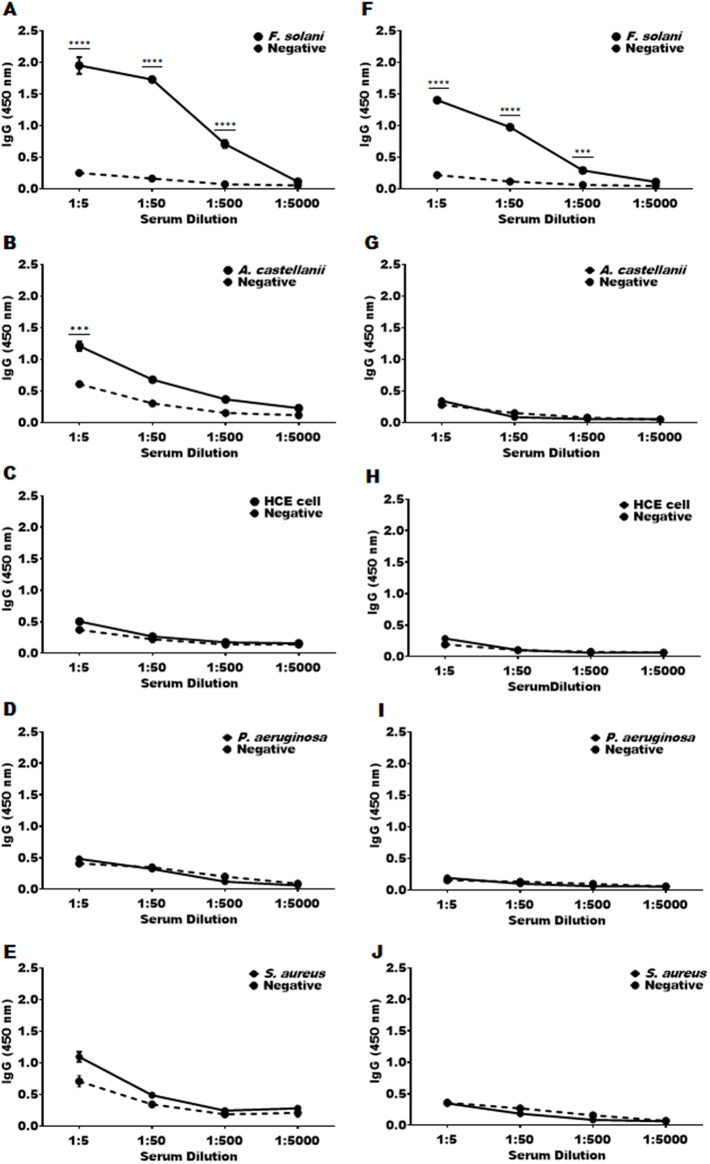
Antibody titration of *F. solani*-specific anti-cutinase polyclonal antibody. The cutinase antibody was titrated through serial dilutions ranging from 1:5 to 1:5,000, using cell lysates (A-E) and conditioned media (F-J) from various organisms: A and F represent *F. solani*, B and G represent *A. castellanii*, C and H represent HCE cells, D and I represent *P. aeruginosa*, and E and J represent *S. aureus*. Positive groups (bold black line) consisted of immune mouse sera, while negative controls (dotted black line) consisted of naïve mouse sera. Statistical significance between the absorbance values of positive and negative sera at respective dilutions was indicated by asterisks (*** *P* < 0.001 and **** *P* < 0.0001).

To evaluate the response and titer of the cutinase specific antibody, an enzyme-linked immunosorbent assay (ELISA) was conducted utilizing cell lysates (Fig 2A–E) and conditioned media (Fig 2F–J) derived from *F. solani*, *A. castellanii*, human corneal epithelial (HCE) cells, *P. aeruginosa*, and *S. aureus*. The antigens at a concentration of 10 μg/ml were coated on a 96-well plate. The serum was then subjected to serial dilutions, ranging from 1:5–1:5,000, with each step representing a 10-fold reduction. The IgG specific to cutinase was detectable at dilutions as high as 1:500 in both the cell lysate (Fig 2A) and conditioned media (Fig 2F) of *F. solani*, with no reactivity observed towards other organisms (Fig 2C–E, G–J), except for a notable reaction at a 1:5 dilution with *A. castellanii* (Fig 2B).

**Fig 3:**
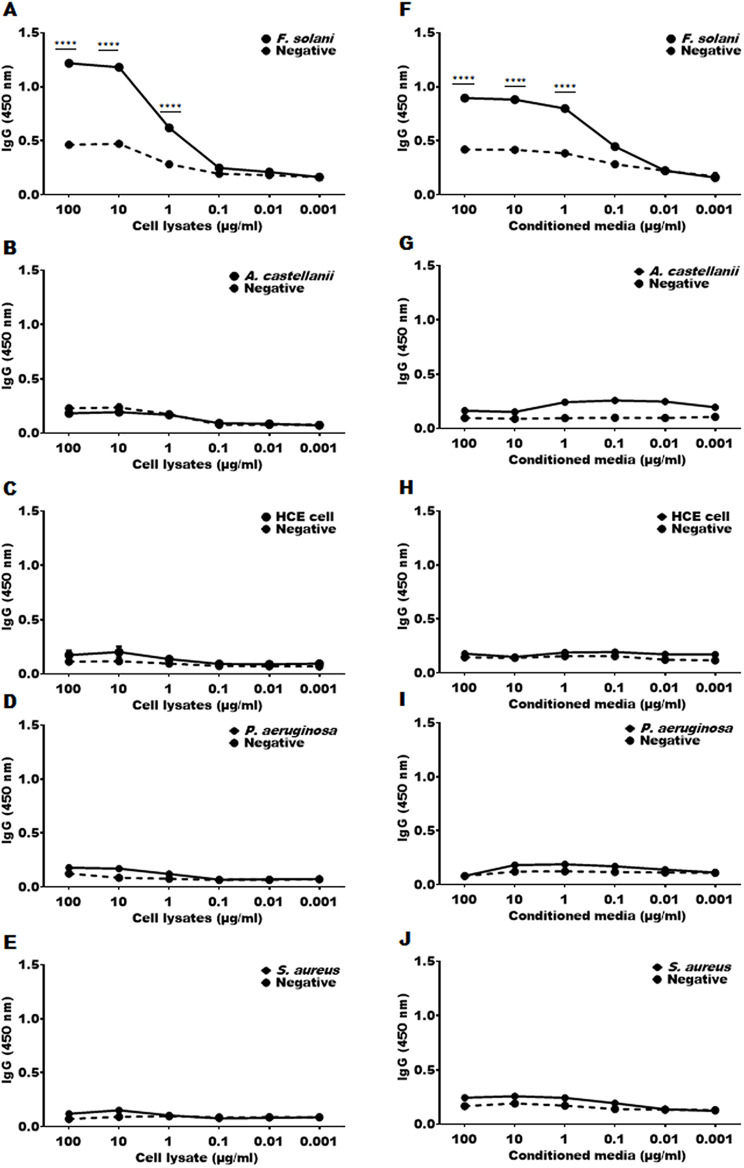
Assessing the sensitivity of *F. solani* anti-cutinase polyclonal antibody. The sensitivity of the cutinase antibody was assessed using serial dilutions of cell lysates (A-E) and conditioned media (F-J), ranging from 100 to 0.001 μg/ml. Positive groups (bold black line) consisted of immune mouse sera, while negative controls (dotted black line) consisted of naïve mouse sera. Specifically, A and F correspond to *F. solani*, B and G to *A. castellanii*, C and H to HCE cells, D and I to *P. aeruginosa*, and E and J to *S. aureus*. Statistical significance between the absorbance values of positive and negative sera at respective dilutions was indicated by asterisks (*** *P* < 0.001 and **** *P* < 0.0001).

To assess the sensitivity of the cutinase specific antibody, the cell lysate (Fig 3A–E) and conditioned media (Fig 3F–J) were serially diluted from 100 µg/ml to 0.001 µg/ml and coated. The serum was then applied at a concentration of 1:50. Detection was successfully achieved down to a concentration of 1 µg/ml in both the cell lysate (Fig 3A) and conditioned media (Fig 3F) of *F. solani*. No reactivity was observed with other organisms (Fig 3B–E, G–J), thereby confirming the specificity of the antibody for *F. solani*.

### Assessment of cutinase antibody specificity for *F. solani*

**Fig 4:**
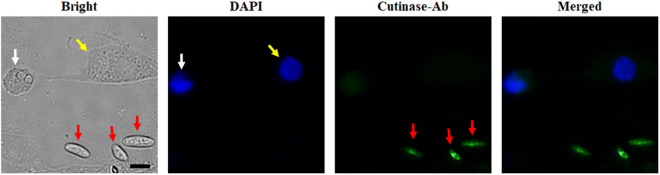
Immunocytochemistry using the *F. solani* cutinase antibody. HCE cells were co-cultured with *F. solani*, *A. castellanii*, *P. aeruginosa*, and *S. aureus*. The white arrow indicates *A. castellanii*, the yellow arrow indicates HCE cell, and the red arrows indicate *F. solani*. The co-cultured cells were subsequently incubated with a cutinase-specific antibody followed by a CFL488-labeled anti-mouse IgG secondary antibody (green). *Acanthamoeba* and HCE cell were counterstained with DAPI (blue) prior to fluorescence microscopy. Bright-field, DAPI, cutinase-antibody, and merged images were captured at 400X magnification. The black scale bar represents 10 μm.

To confirm the specificity of the cutinase antibody, immunocytochemistry was employed using HCE cells co-cultured with *F. solani*, *A. castellanii*, *P. aeruginosa*, and *S. aureus*. The cutinase antibody specifically identified *F. solani*, as indicated by the green fluorescence (denoted by red arrows), while no staining was observed for the other organisms (Fig 4). The nuclei of HCE cells (yellow arrow) and *A. castellanii* (white arrow) were counterstained with DAPI, resulting in blue fluorescence. These findings demonstrate that the cutinase antibody possesses specific detection capabilities for *F. solani*.

### Detection of *Fusarium* antigens in an FK mouse model using cutinase antibody

**Fig 5:**
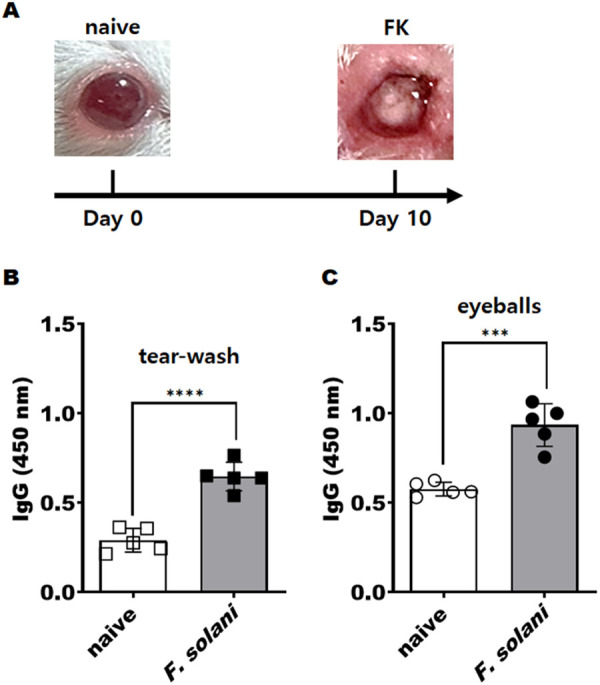
Detection of *Fusarium* antigens in FK mouse model samples using cutinase antibody. Fungal keratitis was induced in BALB/c mice by exposing the scratched cornea of each mouse to contact lenses infected with *F. solani* for 10 days. Compared to naïve mice, typical inflammation and symptoms of keratitis were observed in *Fusarium*-infected mice (A). *Fusarium* antigens were detected by ELISA assay using cutinase-specific antibodies in both tear-wash samples (B) and sonicated eyeballs in PBS (C). Negative controls (□) correspond to naïve tear-wash samples, and positive groups (■) correspond to FK-mouse tear-wash samples. Similarly, negative controls (○) correspond to naïve eyeball lysates, and positive groups (●) correspond to FK-mouse eyeball lysates. Asterisks indicate statistical significance between the means (*** *P* < 0.001 and **** *P* < 0.0001).

To assess the antigen detection capability of the cutinase antibody, a fungal keratitis (FK) mouse model was established. Symptoms of FK were observed on the 10th day following infection with *F. solani* (Fig 5A). The specificity of the cutinase antibody for *Fusarium* was assessed in vivo via ELISA, utilizing tear-wash samples and whole eyeball lysates obtained from the FK mouse model. The cutinase antibody exhibited specific reactivity with both tear-wash samples and whole eyeball lysates (Fig 5B, C). These findings confirm that the cutinase antibody is capable of detecting *Fusarium* antigens within the FK mouse model.

## Discussion

Fungal keratitis (FK) can result in blindness and enucleation if not diagnosed and treated promptly, underscoring the importance of rapid and accurate diagnosis to prevent severe outcomes [18]. In this study, we generated a polyclonal antibody against the cutinase protein of *F. solani* and evaluated its potential for the rapid and accurate diagnosis of FK. The cutinase antibody successfully detected *F. solani* antigens in tear-wash samples and whole eyeball lysates from the FK mouse model.

The current standard diagnostic methods for FK, including culture and microscopic examination, necessitate corneal scrapes or biopsies. These procedures are often painful for patients and time-intensive. Consequently, the development of a non-invasive, rapid, and straightforward diagnostic approach is warranted [19]. In this study, the cutinase antibodies were found to significantly detect fungal antigens responsible for keratitis in the tears of an animal model with FK (Fig 5B). This finding suggests the potential for diagnosing FK by identifying the causative antigens in a patient’s tears, eliminating the need for corneal scraping. Additionally, as this method does not require culturing, it can significantly reduce the diagnostic time.

However, the sensitivity of the antigen-antibody reaction observed in tear samples from animal models of FK is low, and the antigen may be diluted by tears, indicating the need for further research to enhance the sensitivity of the reaction. In previous studies, the use of chorismate mutase (CM) antibodies to detect *Acanthamoeba* antigens in the tears of patients with *Acanthamoeba* keratitis demonstrated significantly increased sensitivity of the antigen-antibody reaction when utilizing a Surface-Enhanced Raman Scattering (SERS) platform [20]. This platform combined Ag@AuFNP with CM-specific antibody complexes. If the cutinase antibodies are employed within such a platform, it is anticipated that the detection sensitivity for antigens in the diagnosis of FK could be significantly enhanced. By integrating technologies such as SERS with cutinase antibodies, it is possible to augment the sensitivity of the antigen-antibody reaction, potentially leading to more effective and rapid diagnostic methods for FK.

The cutinase antibody exhibited a significant reaction with both the cell lysate and conditioned media from *F. solani* (Figs 2 and 3). However, it also demonstrated a reaction with the cell lysate from *A. castellanii* (Fig 2B). Although the cutinase fungal antibodies exhibited reactivity with *Acanthamoeba* cell lysate during ELISA testing, immunocytochemistry (ICC) results demonstrated that these antibodies did not react with *A. castellanii*, but specifically targeted *F. solani* (Fig 4). This suggests that while ELISA results indicated potential cross-reactivity in a cell lysate context, ICC confirmed the antibodies’ specificity to fungal cells in a cellular context. These results suggest the possibility of specifically detecting *F. solani* using cutinase antibodies when *A. castellanii*, *F. solani*, and bacteria are co-infected in corneal cells.

In this study, the cutinase antibody showed significant reactivity with *F. solani*. However, its reactivity with other fungal species, such as *Aspergillus* spp. and *Candida* spp., has not been evaluated. In addition to these findings, further experiments are necessary to investigate whether cutinase antibodies can also detect other species of *Fusarium*. Furthermore, antigen detection evaluations should be carried out on tear samples from other corneal ulcer animal models, including those caused by different types of fungal keratitis, *Acanthamoeba* keratitis, and bacterial keratitis. Conducting these evaluations would aid in assessing the specificity and effectiveness of the cutinase antibodies in differentiating between various forms of keratitis and ensuring accurate diagnosis across a range of infectious agents.

In summary, the polyclonal antibody against the cutinase protein of *F. solani* produced in this study confirms its ability to selectively detect *F. solani* antigens. This indicates its promising potential for application in the development of diagnostic tools for fungal keratitis, enabling more accurate and specific detection of this pathogen.
